# Data on a high electrocatalytic activity of metal-WO_3_ nanocomposite electrocatalysts for hydrogen evolution reaction

**DOI:** 10.1016/j.dib.2023.109362

**Published:** 2023-07-03

**Authors:** Ngoc-Anh Nguyen, Enkhjin Chuluunbat, Ho-Suk Choi, Michael Keidar

**Affiliations:** a Department of Chemical Engineering and Applied Chemistry, Chungnam National University, 99 Daehak-ro, Yuseong-Gu, Daejeon 34134, Republic of Korea; b Department of Mechanical and Aerospace Engineering, The George Washington University, Washington DC 20052, USA

**Keywords:** Metal-WO_3_ catalysts, Hydrogen evolution reaction, Water splitting, Hydrogen spillover effect

## Abstract

The data given in this article are related to the research article entitled “High electrocatalytic activity of Rh-WO_3_ electrocatalyst for hydrogen evolution reaction under the acidic, alkaline, and alkaline seawater electrolytes (N.-A. Nguyen et al., 2023) [1]. In this work, metal-WO_3_ nanocomposites were synthesized and used as electrocatalysts for hydrogen evolution reaction (HER) performance. The morphology and chemical properties of the prepared metal-WO_3_ nanocomposites were investigated by using scanning electron microscopy (SEM), and X-ray photoelectron spectroscopy (XPS) techniques.

**Specifications Table**

**Table:** 

Subject	Chemical Engineering: Catalysis

Specific subject area	Synthesis of catalysts for hydrogen evolution reaction
Type of data	Table and Figures
How the data were acquired	The morphology of materials was investigated by using scanning electron microscopy (SEM, FE-SEM, Hitachi S-4800, Japan) (Accelerating volatage:0.5-30kV, Resolution: 1.0nm 15kV, Magnification: x25-x800,000, Electron gun: Cold-cathode field emission type electron gun). X-ray photoelectron spectroscopy (XPS) data of materials were recorded by using a Thermo Fisher Theta Probe system equipped with a monochromated Al-K X-ray source (K-Alpha+, Thermo Fisher Scientific) (Type: Spherical sector analyzer, Mean diameter:250mm, Operation mode: CAE, Min. energy step size: 3 meV, Ultimate Energy resolution (of Ag 3d5/2 peak): <0.5eV FWHM, Ultimate spatial resolution: <30 µm, by knife edge method, XPS on Insulators: C1s energy resolution (eV): <0.85eV, Pass Energy:1 - 400 eV, Analyser Housing: Mu-metal shielded, Detector:128-channel detector, Max. Sensitivity at 1eV FWHM on Ag3d5, at 400 µm:4,000,000 cps). HER performance was collected by using a 3-electrode cell and a Potentiostat (IviumStat).
Data format	Raw and Analyzed
Description of data collection	The metal-WO_3_ materials were fabricated by the chemical reduction method. The prepared metal-WO_3_ materials were dispersed in absolute ethanol and deposited on a silicon wafer for SEM and XPS measurements. The HER performance data was collected by measuring linear sweep voltammetry (LSV) using a 3-electrode system and a Potentiostat (IViumStat) with a scan rate of 10 mV/s in the acidic electrolyte.
Data source location	Center for Research Facilities and Department of Chemical Engineering and Applied Chemistry, Chungnam National University, Daejeon, South Korea
Data accessibility	https://figshare.com/s/b0531071d8741fae6fda or DOI number: 10.6084/m9.figshare.23354159
Related research article	Ngoc-Anh Nguyen, Enkhjin Chuluunbat, Tuan Anh Nguyen, Ho-Suk Choi, “High electrocatalytic activity of Rh-WO_3_ electrocatalyst for hydrogen evolution reaction under the acidic, alkaline, and alkaline seawater electrolytes” International Journal of Hydrogen Energy DOI number: https://doi.org/10.1016/j.ijhydene.2023.05.067

## Value of the Data

• Metal-WO_3_ nanocomposites were synthesized by using ethylene glycol/ NaBH_4_ as a reducing agent.
• SEM images are shown to see the morphology of metal-WO_3_ nanocomposites electrocatalysts synthesized.
• XPS technique was used to understand the electronic structure of electrocatalysts synthesized.
• Electrochemical tests of metal-WO_3_ electrocatalysts were performed to see their excellent hydrogen evaluation reaction (HER) performance.

## 1 Objective

The data provide more information about the synthesis of metal-WO_3_ nanocomposites and their application as electrocatalysts for the hydrogen evolution reaction in the acidic electrolyte. SEM and XPS data show evidence of success in the synthesis of metal-WO_3_ nanocomposites. Values collected from electrochemical performance confirm that the metal-WO_3_ nanocomposites are highly promising HER electrocatalysts in hydrogen production by water splitting.

## 2 Data Description

**Fig. 1:**
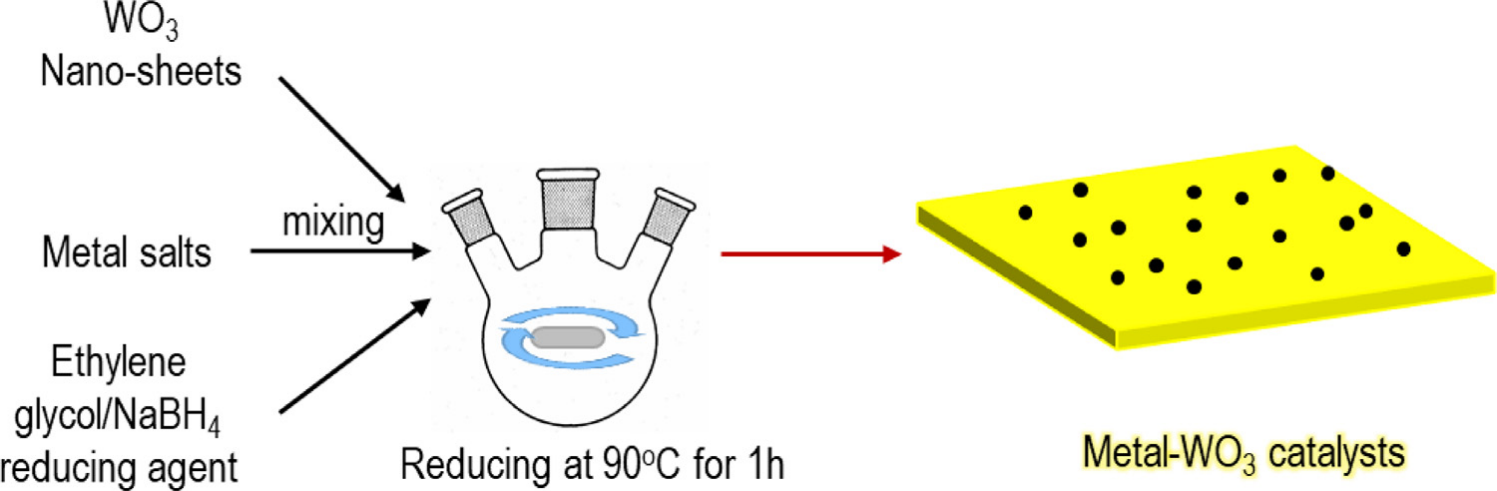
The illustration of the fabrication of materials.

**Fig. 2:**
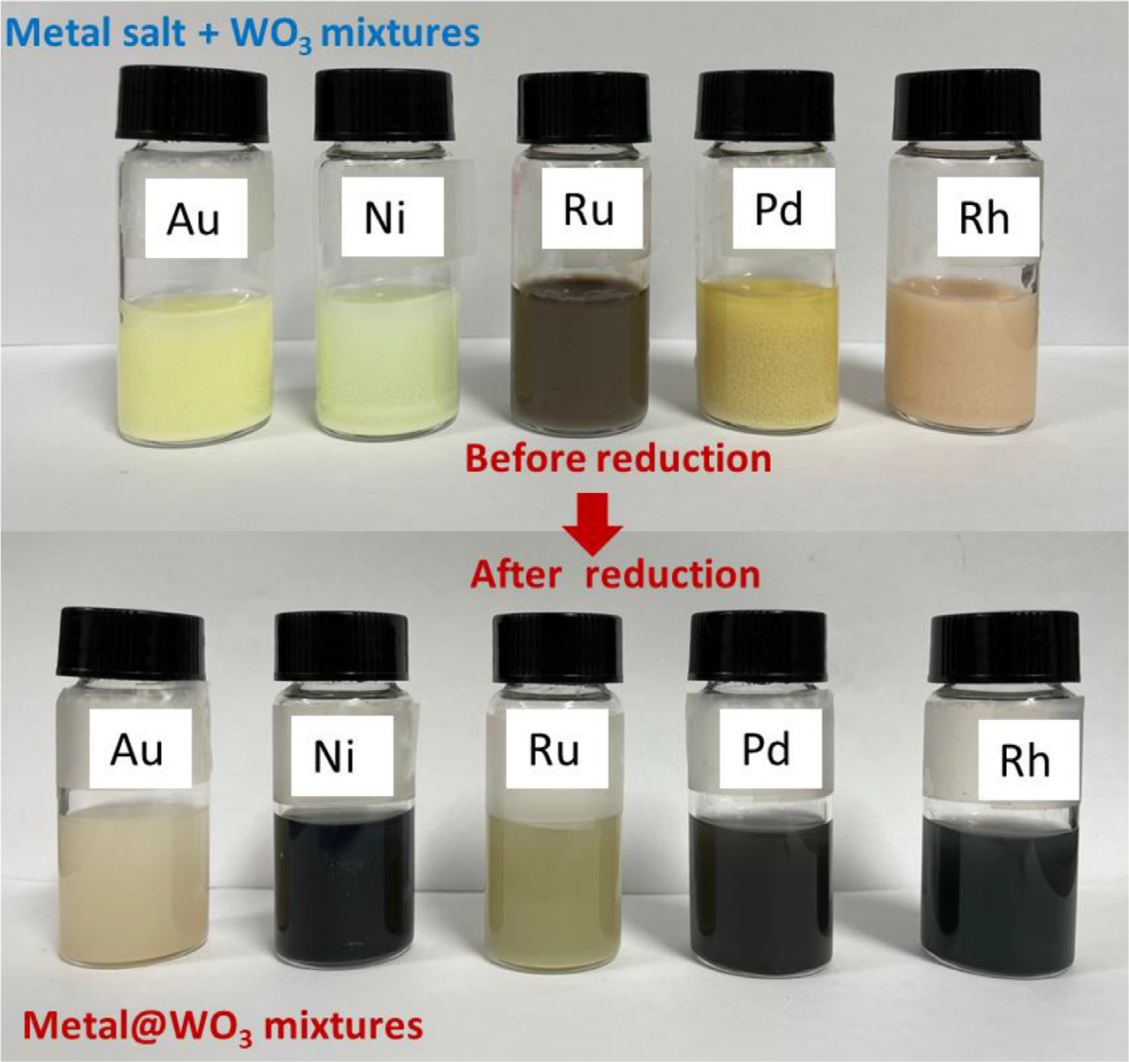
The digital images of the precursor solutions before and after the reaction of the synthesis.

**Fig. 3:**
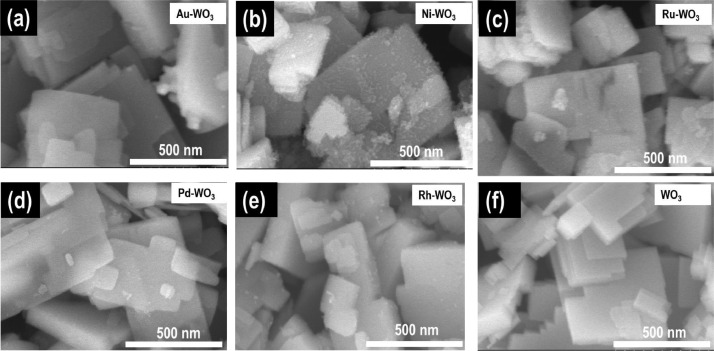
SEM images of synthesized metal-WO_3_ materials. (a) Au-WO_3_, (b) Ni-WO_3_, (c) Ru-WO_3_, (d) Pd-WO_3_, (e) Rh-WO_3_, (f) bare WO_3_, respectively.

**Fig. 4:**
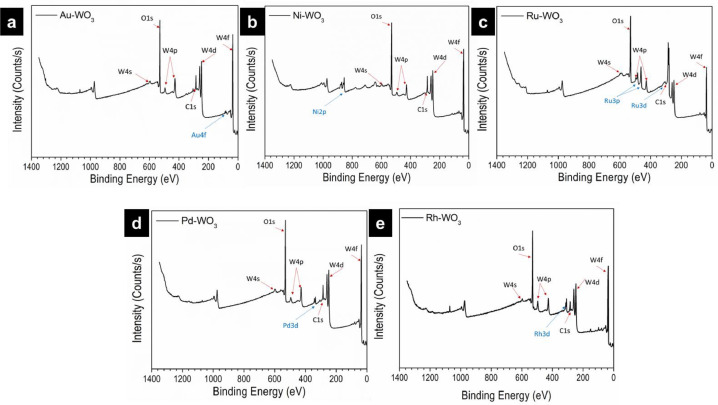
The XPS survey spectra of synthesized metal-WO_3_ materials. (a) Au-WO_3_, (b) Ni-WO_3_, (c) Ru-WO_3_, (d) Pd-WO_3_, (e) Rh-WO_3_, respectively.

The data provide characterizations of prepared metal-WO_3_ nanocomposites such as their morphology, electronic structure, and HER catalytic activity. Water splitting is a good way to hydrogen production [1], [2], [3], [4], [5]. Currently, WO_3_-based materials are considered a very potential HER candidate catalyst [6], [7]. Herein, we report a simple method to prepare metal-WO_3_ materials and use them as hydrogen evolution electrocatalysts. The synthesis process of metal-WO_3_ materials was illustrated in Fig. 1. The different colors before and after the synthesis of the precursor solution can be seen in Fig. 2. The prepared metal-WO_3_ materials were investigated morphology by SEM pictures as seen in Fig. 3. The XPS spectra were taken to investigate the electronic structure of all prepared metal-WO_3_ materials (Fig. 4). In addition, the HER activities of metal-WO_3_ catalysts in other works are summarized as can be seen in Table 1.

**Table 1:** Electrochemical performance data for Rh-WO_3_, Pd-WO_3_, Au-WO_3_, Ru-WO_3_, Ni-WO_3_ and commercial Pt/C catalysts compared with metal-only catalysts in previous works.

Electrocatalysts	Overpotential (mV) at j= -10mA.cm^−2^	Metal loading (wt%)	Ref.
Rh-WO_3_	48	5	This work
Pd-WO_3_	141	5	This work
Au-WO_3_	273	5	This work
Ru-WO_3_	192	5	This work
Ni-WO_3_	456	5	This work
WO_3_	551	5	This work
Pt/C	45	20	This work
Ni	403	100	[9]
Ni	450	100	[10]
Au	180	100	[11]
Au	>350	100	[12]
Pd	20	100	[13]
Pd	55	100	[14]
Ru-GLC	35	62	[15]
Rh	109	100	[9]
Rh	233	100	[16]

All metal-WO_3_ catalysts were successfully synthesized and confirmed by the color change of the precursor solution before and after the reaction. Clearly, metal nanoparticles (Pd, Au, Ru, Ni, and Rh) were deposited on WO_3_ nanosheets as displayed in SEM images (Fig. 3). The XPS spectra are evidence that metal nanoparticles were deposited on WO_3_ nanosheets as seen in Fig. 4. In more detail, the fitted peaks of the Rh attributed to metallic Rh at 531 eV and 532.4 eV, suggesting that the Rh nanoparticle was successfully deposited on WO_3_ nanosheets [1].

In addition, the HER activity of the prepared catalyst was recorded following the work reported [8]. From the HER performance collected, we can see the benefits of the hydrogen spillover effect of prepared metal-WO_3_ catalysts by comparing their HER activity with metal-only catalysts in previous work (Table 1) [9], [10], [11], [12], [13], [14], [15], [16]. Clearly, all prepared metal-WO_3_ catalysts show higher HER activity compared to those of metal-only catalysts. Among prepared catalysts, Rh-WO_3_ (5%wt of Rh) shows the best HER activity with the overpotential value (48 mV), which is similar to that of the commercial Pt/C (20wt% of Pt) (45 mV). The main reason comes from the hydrogen spillover effect [1,6,7]. Thus, the low metal amount along with the hydrogen spillover effect can improve the HER activity of electrocatalysts for water splitting to produce sustainable hydrogen.

## 3 Experimental Design, Materials and Methods

### 3.1 Materials

All chemicals used in this study including NiCl_2_.6H_2_O (99,999% trace metal basic), HAuCl_4_, (99.999% trace metals basis), RuCl_3_·xH_2_O (99.98% trace metals basis), K_2_PdCl_4_ (99.99% trace metals basis), ethylene glycol (anhydrous, 99.8%), NaBH_4_ powder (≥99.0%), and Sulfuric acid (H_2_SO4, 95-98%) were provided by Sigma Aldrich (USA).

### 3.2 Methods

The synthesis processes of Pd-WO_3_, Au-WO_3_, and Ru-WO_3_ materials are similar to the synthesis process of the Rh-WO_3_ catalyst [1] (see Fig. 1). Note that Ru-WO_3_ was synthesized at 160 °C for 3 hours. The weight percentage of metals in synthesized materials is fixed at 5wt%. In addition, for preparing Ni-WO_3_, the synthesis process is the same as for obtaining the above materials except for adding slowly 10 mL 0.1M NaBH_4_ solution. Then, all prepared metal-WO_3_ materials were analyzed for their characterizations and HER catalytic activity.

### 3.3 Experimental Design

First, metal-WO_3_ materials are synthesized and then their physical characteristics including morphology and surface chemical state are analyzed by using scanning electron microscopy (SEM) and X-ray photoelectron spectroscopy (XPS) techniques, respectively. Scanning electron microscopy (FE-SEM, Hitachi S-4800) was used to analyze the morphology of the synthesized metal-WO_3_ materials. To analyze the electronic structure of prepared samples, XPS spectra are recorded by using Thermo Fisher Theta Probe equipment.

The HER activity of metal-WO_3_ catalysts was recorded in 0.5 M H_2_SO_4_ electrolyte by using a 3-electrode system and a potentiostat (IviumStats). The linear sweep voltammetry (LSV) curves were recorded with a scan rate of 10 mV/s at room temperature as described somewhere else [8].

## Ethics Statements

The data resulted from experimental neither on animal models nor with human volunteers.

## CRediT authorship contribution statement

**Ngoc-Anh Nguyen:** Conceptualization, Methodology, Investigation, Writing – review & editing. **Enkhjin Chuluunbat:** Conceptualization, Methodology, Investigation, Writing – original draft. **Ho-Suk Choi:** Conceptualization, Methodology, Validation, Formal analysis, Writing – review & editing, Supervision, Project administration, Funding acquisition. **Michael Keidar:** Validation, Writing – review & editing.

## Declaration of Competing Interest

The authors declare that they have no known competing financial interests or personal relationships that could have appeared to influence the work reported in this paper.

## Data Availability

Data on a high electrocatalytic activity of metal-WO3 nanocomposite electrocatalysts for hydrogen evolution reaction (Original data) (Figshare)

## References

[1] Nguyen N.A., Chuluunbat E., Nguyen T.A., Choi H.S. (2023 May 20). High electrocatalytic activity of Rh-WO_3_ electrocatalyst for hydrogen evolution reaction under the acidic, alkaline, and alkaline-seawater electrolytes. International Journal of Hydrogen Energy.

[2] Terlouw T., Bauer C., McKenna R., Mazzotti M. (2022). Large-scale hydrogen production via water electrolysis: a techno-economic and environmental assessment. Energy & Environmental Science.

[3] Nguyen N.A., Ali Y., Nguyen V.T., Omelianovych O., Larina L.L., Choi H.S. (2020 Dec 30). NiCoPt/graphene-dot nanosponge as a highly stable electrocatalyst for efficient hydrogen evolution reaction in acidic electrolyte. Journal of Alloys and Compounds.

[4] Nguyen N.A., Le T.H., Trinh V.H., Ngo Q.T., Nguyen V.T., Lee G., Choi H.S., Chen G. (2021 Apr 12). Au/Cdot-Nanohybrid Electrocatalyst Synthesized by Rice-Straw-Derived Carbon Dots as a Reducing Agent for Improved Hydrogen Evolution Reactions. Journal of The Electrochemical Society.

[5] Lee G., Nguyen N.A., Nguyen V.T., Larina L.L., Chuluunbat E., Park E., Kim J., Choi H.S., Keidar M. (2022 Oct 1). High entropy alloy electrocatalyst synthesized using plasma ionic liquid reduction. Journal of Solid State Chemistry.

[6] Cho J., Kim M., Seok H., Choi G.H., Yoo S.S., Sagaya Selvam N.C., Yoo P.J., Kim T. (2022 May 13). Patchwork-Structured Heterointerface of 1T-WS_2_/a-WO_3_ with Sustained Hydrogen Spillover as a Highly Efficient Hydrogen Evolution Reaction Electrocatalyst. ACS Applied Materials & Interfaces.

[7] Li Y., Zhai X., Liu Y., Wei H., Ma J., Chen M., Liu X., Zhang W., Wang G., Ren F., Wei S. (2020 May 15). WO_3_-based materials as electrocatalysts for hydrogen evolution reaction. Frontiers in Materials.

[8] Nguyen V.T., Lee G.J., Ngo Q.T., Omelianovych O., Nguyen N.A., Trinh V.H., Choi H.S., Mnoyan A., Lee K., Larina L.L., Chen G. (2021 Dec 1). Robust carbon-encapsulated Ni nanoparticles as high-performance electrocatalysts for the hydrogen evolution reaction in highly acidic media. Electrochimica Acta.

[9] Nguyen N.A., Nguyen V.T., Shin S., Choi H.S. (2019 Jun 15). NiRh nanosponges with highly efficient electrocatalytic performance for hydrogen evolution reaction. Journal of Alloys and Compounds.

[10] Xu Y., Yin S., Li C., Deng K., Xue H., Li X., Wang H., Wang L. (2018). Low-ruthenium-content NiRu nanoalloys encapsulated in nitrogen-doped carbon as highly efficient and pH-universal electrocatalysts for the hydrogen evolution reaction. Journal of Materials Chemistry A.

[11] Tran T.D., Nguyen M.T., Le H.V., Nguyen D.N., Truong Q.D., Tran P.D. (2018). Gold nanoparticles as an outstanding catalyst for the hydrogen evolution reaction. Chemical Communications.

[12] Wang Y., Sun Y., Liao H., Sun S., Li S., Ager J.W., ZJ Xu (2016 Aug 10). Activation effect of electrochemical cycling on gold nanoparticles towards the hydrogen evolution reaction in sulfuric acid. Electrochimica Acta.

[13] Grigoriev S.A., Millet P., Fateev VN. (2008 Mar 1). Evaluation of carbon-supported Pt and Pd nanoparticles for the hydrogen evolution reaction in PEM water electrolysers. Journal of Power Sources.

[14] Bhowmik T., Kundu M.K., Barman S. (2016 Mar 4). Palladium nanoparticle–graphitic carbon nitride porous synergistic catalyst for hydrogen evolution/oxidation reactions over a broad range of pH and correlation of its catalytic activity with measured hydrogen binding energy. Acs Catalysis.

[15] Chen Z., Lu J., Ai Y., Ji Y., Adschiri T., Wan L. (2016 Dec 28). Ruthenium/graphene-like layered carbon composite as an efficient hydrogen evolution reaction electrocatalyst. ACS Applied Materials & Interfaces.

[16] Cheng Y., Lu S., Liao F., Liu L., Li Y., Shao M. (2017 Jun). Rh-MoS_2_ Nanocomposite Catalysts with Pt-Like Activity for Hydrogen Evolution Reaction. Advanced Functional Materials.

